# Supplementary material for: Paired immunoglobulin-like receptor B is an entry receptor for mammalian orthoreovirus
Source: Nat Commun. 2023 May 5;14:2615. doi: 10.1038/s41467-023-38327-6 (PMC10163058; doi:10.1038/s41467-023-38327-6)
Supplement: Supplementary file 1 — Supplementary Information [file 41467_2023_38327_MOESM1_ESM.pdf]

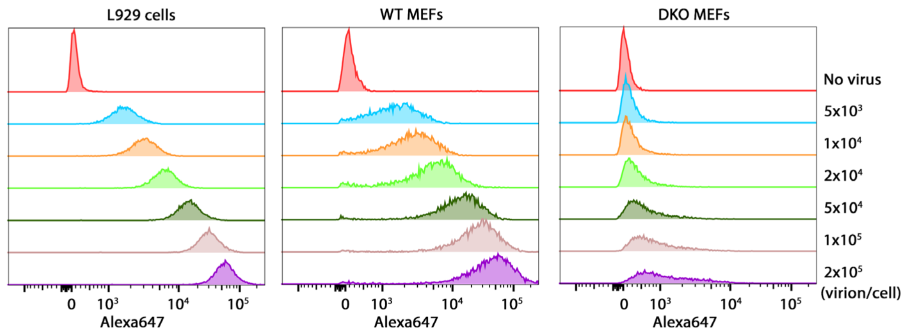

Supplemental figure 1 JAM-A$^{-/-}$ x NgR1$^{-/-}$ MEFs exhibit weak reovirus binding capacity. The cell lines shown were incubated with escalating concentrations of Alexa-647-labelled T3SA- virions. Reovirus binding was measured by flow cytometry and analyzed using FlowJo software. Representative histograms are shown. WT MEFs and L929 cells expressing abundant JAM-A and were used as positive controls.

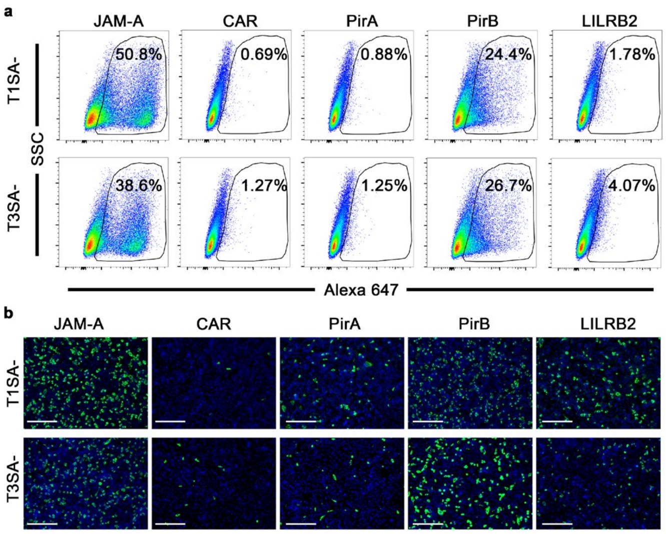

Supplemental figure 2 PirB promotes reovirus binding and infection. a Reovirus binding to receptor-expressing cells. CHO cells transfected with the receptor-encoding cDNAs were adsorbed with Alexa-647-labelled T1SA- or T3SA- virions and analyzed for binding using flow cytometry. cDNAs encoding JAM-A and CAR were used as positive and negative controls, respectively. Binding results were analyzed and plotted using FlowJo software. Representative scatter dot plots are shown. Boxed area indicates highly fluorescent cells with the percentage shown relative to the total population. **b** Reovirus infection of receptor-expressing cells. CHO cells transfected with the receptor-encoding cDNAs were adsorbed with T1SA- or T3SA- virions at MOIs of 10 or 100 PFU/cell. Infectivity was quantified by IFA. Representative images of cells infected at an MOI of 100 PFU/cell are shown. Reovirus proteins and nuclei are pseudocolored in green and blue, respectively. Error bar, 100 μm. In **a** and **b**, experiments were conducted in quadruplicate and triplicate, respectively. Representative images were shown.

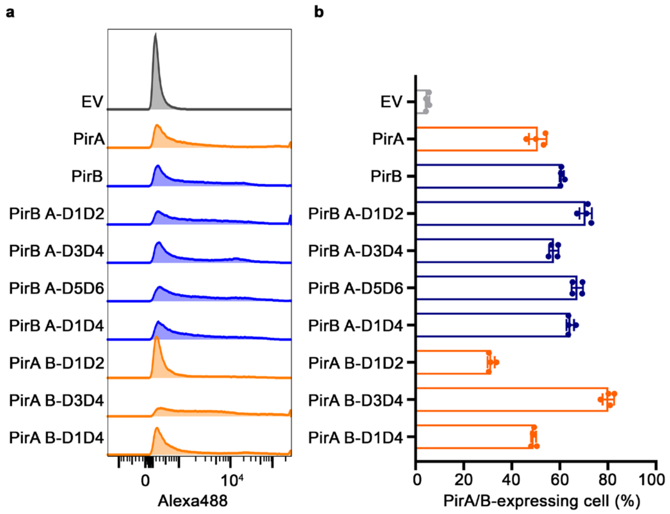

Supplemental figure 3 Expression of chimeric PirA and PirB receptors in CHO cells. CHO cells were transfected with the chimeric receptor cDNAs shown. Empty vector (EV) was used as a negative control. At 48 hpt, cells were incubated with PirA/PirB-specific mAb 6C1, followed by incubation with Alexa-488-labelled rat IgG-specific secondary antibody. Protein expression was quantified using flow cytometry. Data were analyzed using FlowJo software. Representative histograms are shown (**a**). Mean values of receptor-expressing cell percentage are shown (**b**). Experiment was conducted in quadruplicate. Error bars indicated SD.

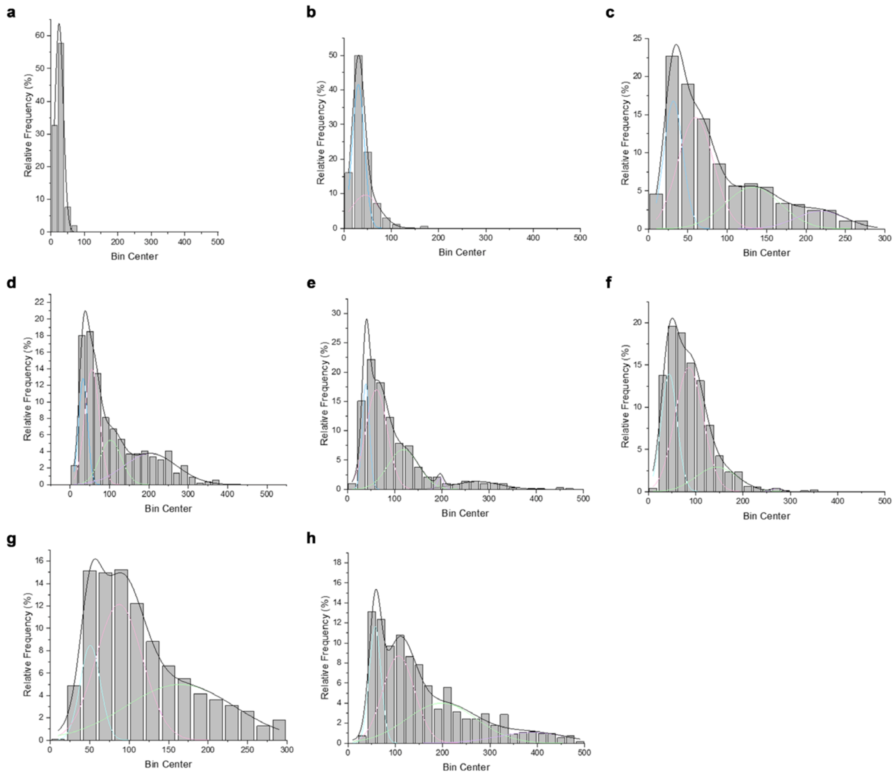

Supplemental figure 4 Histograms and Gaussian fits from each loading rate range used for the dynamic force spectroscopy calculation. The loading rate ranges (pN/Sec) in order are 0-349.9 (**a**); 350-999.9 (**b**); 1000-3499.9 (**c**); 3500-9999.9 (**d**); 10000-34999.9 (**e**); 35000-999999.9 (**f**); 1000000-349999.9 (**g**); and 350000+ (**h**). These loading rate ranges were selected based on previous experiments using reovirus receptors JAM-A and NgR1. Data were fit with the Gaussian Peak Fitting Tool within the Origin Pro software. Peaks were selected based on the shape of the histogram.

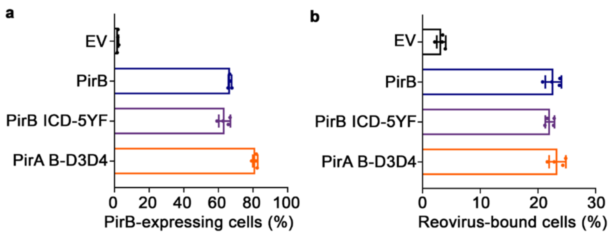

Supplemental figure 5 Reovirus binding to cells expressing PirB intracellular domain mutants. a Expression of WT and mutant PirB in CHO cells. CHO cells were transfected with WT and mutant PirB cDNAs. EV was used as a negative control. Cells were incubated with PE-conjugated PirA/PirB-specific rat mAb 6C1. Protein expression was quantified using flow cytometry. The percentage of PE^{+} cells was analyzed using FlowJo software. **b** Reovirus binding to CHO cells expressing WT and mutant PirB. EV was used as a negative control. CHO cells were adsorbed with Alexa-647-labelled T3SA- virions and scored for reovirus binding using flow cytometry. Binding results were analyzed using FlowJo software. In **a** and **b**, experiments were conducted in quadruplicate. Mean values are shown. Error bars indicate SD.

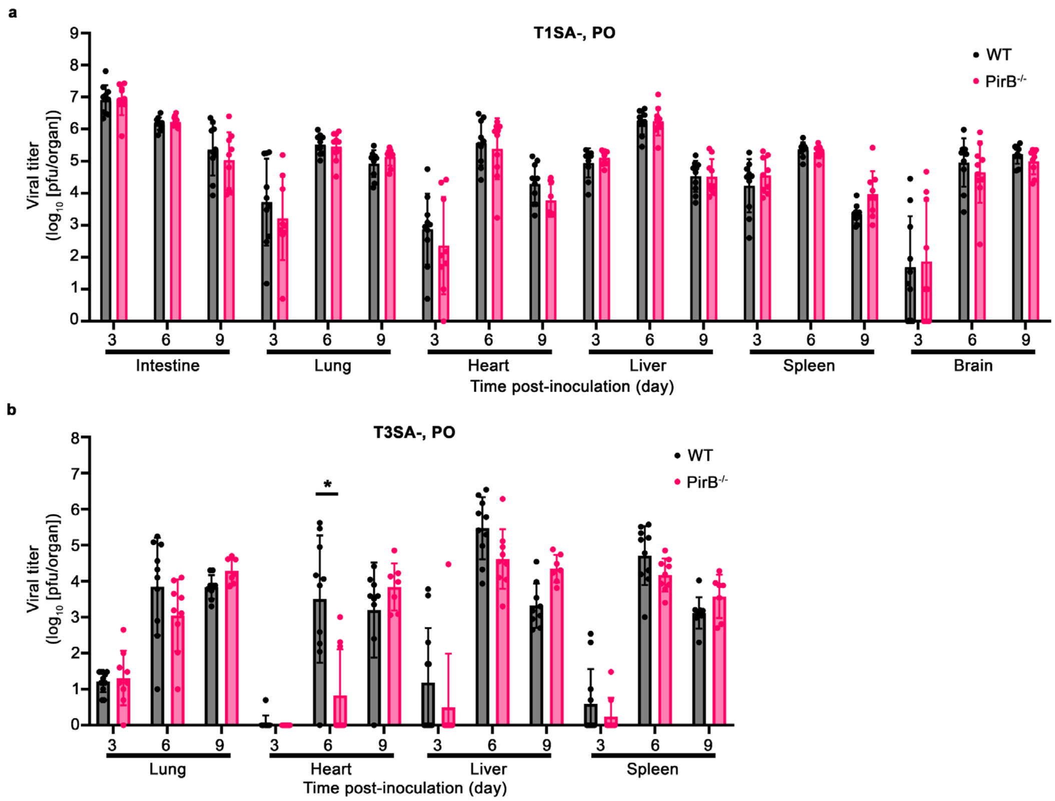

Supplemental figure 6 Function of PirB in reovirus replication in mice. Three-day-old WT and PirB$^{-/-}$ mice were inoculated perorally with 10^4 PFU of T1SA- (**a**) or T3SA- (**b**) reovirus. Mice were euthanized at the dpi shown, and viral titers in organ homogenates were quantified by plaque assay. Each symbol indicates the viral titer determination from a single mouse. In **a**, N=10/10/10 at 3/6/9 DPI (WT); N=9/10/9 at 3/6/9 DPI (PirB$^{-/-}$). In **b**, N=11/10/9 at 3/6/9 DPI (WT); N=9/9/7 at 3/6/9 DPI (PirB$^{-/-}$). Mean values are shown. Error bars indicate SD. Statistical analysis was conducted using two-way ANOVA with Holm-Sidak's test. *, $P < 0.05$.

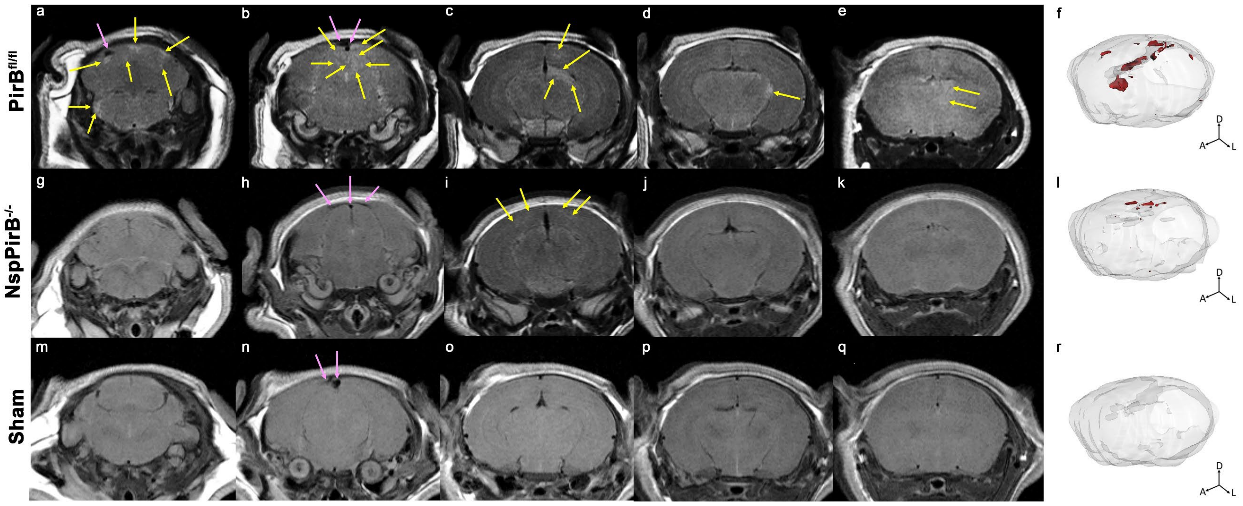

Supplementary figure 7 CNS inflammation evaluation by magnetic resonance imaging. Cerebral inflammation was evaluated by *in vivo* T2-weighted MRI of a $PirB^{fl/fl}$ (**a-f**), $nspPirB^{-/-}$ (**g-l**), and sham control (**m-r**) mouse. (**a-e, g-k, and m-q**) Multi-planar T2-weighted MRI. From left to right: posterior to anterior imaging planes. Yellow arrows indicate cerebral inflammation defined by hyper-intensity, whereas pink arrows pointing to hypo-intensity reflect local hemorrhage from injection. (**f, l, r**) Reconstructed segmented volumes. Red, cerebral inflammation; grey, brain. Orientation axes: A, anterior; D, dorsal; and L, lateral. MRI data were acquired using a 7-Tesla scanner with an in-plane resolution of 70 µm, FOV = 1.8 cm, slice thickness of 0.65 mm, effective TE = 48 msec, and TR = 1551.2 msec.

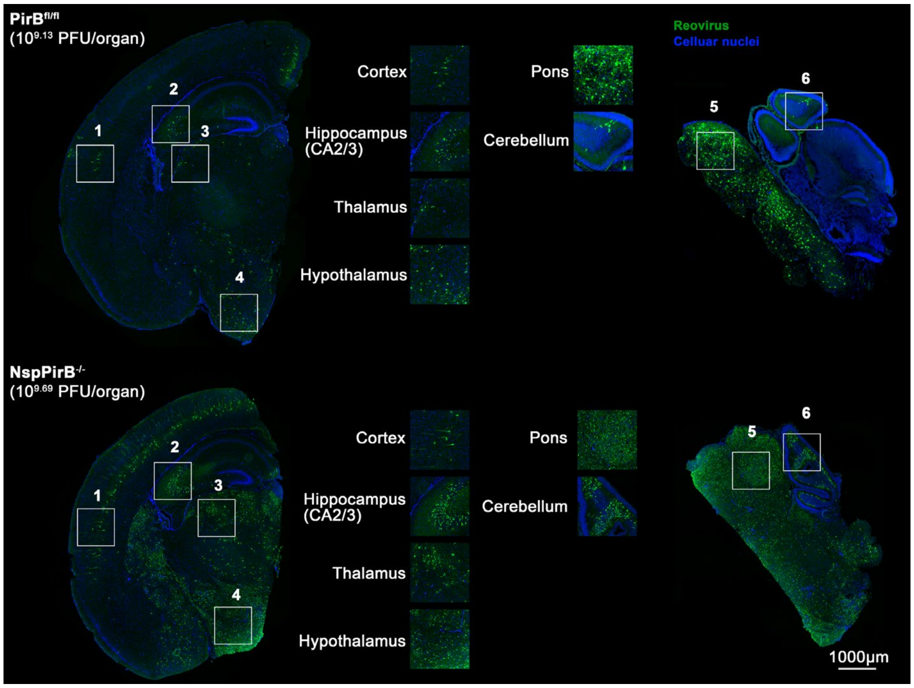

Supplemental figure 8 Reovirus tropism in the brain of PirB$^{fl/fl}$ and NspPirB$^{-/-}$ mice.

Two-day-old PirB$^{fl/fl}$ and NspPirB$^{-/-}$ mice were inoculated intracranially with 25 PFU of T3SA- reovirus. Mice were euthanized at 8 dpi. The right-brain hemisphere was harvested for viral load determination by plaque assay. The left-brain hemisphere was fixed in formalin and embedded in paraffin. To assess for differences in reovirus tropism, coronal sections of the left-brain hemispheres, obtained from brains matched for viral titer, were stained using rabbit polyclonal reovirus-specific antiserum (green). Cellular nuclei were counterstained using DAPI (blue). Representative brain sections show the distribution of reovirus antigen. Enlarged images are derived from the boxed areas in the lower-magnification views and show reovirus infection in regions of cerebral cortex (1), hippocampus (2), thalamus (3), hypothalamus (4), pons (5), and cerebellum (6). Scale bar, 1000 µm.

Supplementary Table 1. Plasmids used in studies of reovirus-PirB interactions

Plasmid	Function	Source	Catalog number
pBSSVD2005	Large T antigen expression	Addgene	21826
dCas9-VP64-blast	CRISPRa transactivation factors expression	Addgene	61425
pCMV-VSV-G	VSV-G expression	Addgene	8454
pxPAX2	Lentivirus packaging vector	Addgene	12260
pXPR-502	CRISPRa sgRNA transfer vector (empty)	Addgene	96923
Mouse CRISPR Activation Pooled Library (Caprano P65-HSF)	CRISPRa library	Addgene	1000000113
pCMV-SPORT6-mouse PirB	PirB cDNA expression	Dharmacon	MMM1013-202766374
pCMV-SPORT6-human LILRB2	LILIB2 cDNA expression	Dharmacon	MHS6278-202801479
pCMV6-entry-mouse PirA	PirA cDNA expression	Origene	MR213778
pcDNA3.1	Mammalian expression vector (empty)	In-house	N/A
pcDNA3.1-hJAM-A	hJAM-A cDNA expression	Previous study[1]	N/A
pcDNA3.1-hCAR	hCAR cDNA expression	Previous study[1]	N/A
pcDNA3.1-hNgR1	hNgR1 cDNA expression	Previous study[2]	N/A
pcDNA3.1-PirB A-D1D2	PirA/B chimera cDNA expression	This paper	N/A

pcDNA3.1-PirB A-D3D4		This paper	N/A
pcDNA3.1-PirB A-D5D6		This paper	N/A
pcDNA3.1-PirB A-D1D4		This paper	N/A
pcDNA3.1-PirA B-D1D2		This paper	N/A
pcDNA3.1-PirA B-D3D4		This paper	N/A
pcDNA3.1-PirA B-D1D4		This paper	N/A

N/A: not applicable.

Supplementary Table 2. Statistical analysis for all figures

Figure	Panel	Test	Comparison	*P*-value	Mean difference	95% confidence intervals
3	b	One-way ANOVA	PirB vs. PirB A-D1D2	<0.0001	-38.48	-42.82 to -34.13
3	b	One-way ANOVA	PirB vs. PirB A-D3D4	<0.0001	14.77	10.42 to 19.11
3	b	One-way ANOVA	PirB vs. PirB A-D1D4	<0.0001	14.72	10.38 to 19.06
3	b	One-way ANOVA	PirA vs. PirA B-D3D4	<0.0001	-46.46	-50.80 to -42.12
3	b	One-way ANOVA	PirA vs. PirA B-D1D4	<0.0001	-11.88	-16.22 to -7.536
3	c	One-way ANOVA	PirB vs. PirB A-D3D4	<0.0001	15.73	7.765 to 23.70
3	c	One-way ANOVA	PirB vs. PirB A-D1D4	<0.0001	15.07	7.099 to 23.03
3	c	One-way ANOVA	PirA vs. PirA B-D3D4	0.0491	-7.733	-15.70 to 0.2347
3	c	One-way ANOVA	PirA vs. PirA B-D1D4	0.0092	-9.867	-17.83 to -1.899
4	b	One-way ANOVA	T3SA- vs T3SA- PirB mAb	<0.0001	11.95338	8.69153 to 15.21523
5	a	One-way ANOVA	0 min vs 10 min	0.0006	-1.475	-2.342 to -0.6070
5	a	One-way ANOVA	0 min vs 15 min	<0.0001	-2.518	-3.386 to -1.651
5	a	One-way ANOVA	0 min vs 20 min	<0.0001	-2.516	-3.383 to -1.648
5	a	One-way ANOVA	0 min vs 25 min	<0.0001	-2.505	-3.373 to -1.638
5	a	One-way ANOVA	0 min vs 30 min	<0.0001	-2.390	-3.258 to -1.523

5	a	One-way ANOVA	0 min vs 60 min	<0.0001	-1.840	-2.708 to -0.9723
5	b	Two-way ANOVA	WT vs 5YF, 3 hpa	0.0002	0.8864	0.3950 to 1.378
5	b	Two-way ANOVA	WT vs B-D3D4, 3 hpa	0.0257	0.5432	0.05176 to 1.035
5	b	Two-way ANOVA	WT vs 5YF, 6 hpa	0.0006	0.7972	0.3057 to 1.289
5	b	Two-way ANOVA	WT vs B-D3D4, 6 hpa	0.0049	0.6618	0.1704 to 1.153
5	b	Two-way ANOVA	WT vs 5YF, 9 hpa	0.0012	0.7583	0.2669 to 1.250
5	b	Two-way ANOVA	WT vs B-D3D4, 9 hpa	0.0011	0.7632	0.2718 to 1.255
5	c	Two-way ANOVA	WT vs 5YF, MOI=20	0.0109	7.500	1.586 to 13.41
5	c	Two-way ANOVA	WT vs B-D3D4, MOI=20	<0.0001	14.55	8.636 to 20.46
5	c	Two-way ANOVA	WT vs B-D3D4, MOI=100	0.0169	7.050	1.136 to 12.96
5	d	Two-way ANOVA	WT vs 5YF, 24 hpa	0.0066	0.2456	0.05964 to 0.4315
5	d	Two-way ANOVA	WT vs B-D3D4, 24 hpa	<0.0001	0.7135	0.5275 to 0.8994
5	d	Two-way ANOVA	WT vs 5YF, 48 hpa	0.0024	0.2734	0.08749 to 0.4593
5	d	Two-way ANOVA	WT vs B-D3D4, 48 hpa	<0.0001	0.5877	0.4017 to 0.7736
5	d	Two-way ANOVA	WT vs B-D3D4, 72 hpa	<0.0001	0.6220	0.4360 to 0.8079
5	e	Two-way ANOVA	WT vs WT inhibitor, 3 hpa	0.0454	0.3450	-0.006102 to 0.6961
5	e	Two-way ANOVA	WT vs 5YF, 3 hpa	0.0006	0.5907	0.2396 to 0.9418
5	e	Two-way ANOVA	WT vs WT inhibitor, 6 hpa	0.0042	0.4870	0.1359 to 0.8381

5	e	Two-way ANOVA	WT vs 5YF, 6 hpa	<0.0001	0.6850	0.3339 to 1.036
5	f	One-way ANOVA	WT vs 5YF, μ1, 10 min	0.0024	-0.2755	-0.4684 to -0.08255
5	f	One-way ANOVA	WT vs 5YF, μ1, 20 min	0.0377	-0.2012	-0.3941 to -0.008290
5	f	One-way ANOVA	WT vs 5YF, μ1, 60 min	<0.0001	-0.4863	-0.6792 to -0.2933
5	f	One-way ANOVA	WT vs 5YF, σ3, 10 min	0.0035	-0.3420	-0.5900 to -0.09403
5	f	One-way ANOVA	WT vs 5YF, σ3, 20 min	0.0090	-0.3095	-0.5575 to -0.06152
5	f	One-way ANOVA	WT vs 5YF, σ3, 30 min	0.0154	-0.2911	-0.5391 to -0.04311
5	f	One-way ANOVA	WT vs 5YF, σ3, 60 min	0.0001	-0.4558	-0.7038 to -0.2078
6	a	Two-way ANOVA	WT vs KO, intestine, 3 hpi	0.0048	0.4351	0.1130 to 0.7572
6	a	Two-way ANOVA	WT vs KO, intestine, 6 hpi	0.0117	-0.4034	-0.7326 to -0.07411
6	a	Two-way ANOVA	WT vs KO, intestine, 9 hpi	0.0246	-0.4021	-0.7632 to -0.04094
6	a	Two-way ANOVA	WT vs KO, brain, 6 hpi	0.0008	2.508	0.9170 to 4.099
6	b	Two-way ANOVA	WT vs KO, brain, 4 hpi	0.0161	1.731	0.2558 to 3.206
6	b	Two-way ANOVA	WT vs KO, brain, 6 hpi	<0.0001	3.846	2.035 to 5.657
6	c	Two-way ANOVA	WT vs KO, brain, 6 hpi	0.0286	3.452	0.3227 to 6.581
6	d	Log-rank (Mantel-Cox) test	WT vs KO	0.0182	N/A	N/A
6	e	Two-way ANOVA	WT vs KO	0.0407	0.2432	0.01034 to 0.4760
6	f	Student’s t-test	Isotype vs mAb	0.0031	75.67 ± 11.88	42.67 to 108.7

6	g	Student’s t-test	WT vs KO	0.0012	50.11 ± 6.081	-66.99 to -33.23
6	h	Two-way ANOVA	WT vs KO, 24 hpa	<0.0001	0.6125	0.4317 to 0.7933
6	h	Two-way ANOVA	WT vs KO, 48 hpa	<0.0001	0.5073	0.3265 to 0.6881
6	h	Two-way ANOVA	WT vs KO, 72 hpa	0.0006	0.3435	0.1627 to 0.5243
S6	b	Two-way ANOVA	WT vs KO, heart, 6 hpi	<0.0001	2.670	1.436 to 3.903

NA: not applicable.

Reference

1 Barton, E. S. *et al.* Junction adhesion molecule is a receptor for reovirus. *Cell* **104**, 441-451 (2001).

2 Konopka-Anstadt, J. L. *et al.* The Nogo receptor NgR1 mediates infection by mammalian reovirus. *Cell Host Microbe* **15**, 681-691 (2014).
