# Supplementary material for: Paired immunoglobulin-like receptor B is an entry receptor for mammalian orthoreovirus
Source: Nat Commun. 2023 May 5;14:2615. doi: 10.1038/s41467-023-38327-6 (PMC10163058; doi:10.1038/s41467-023-38327-6)
Supplement: Supplementary file 3 — Reporting Summary [file 41467_2023_38327_MOESM3_ESM.pdf]

nature portfolio

Corresponding author(s): Terence S. Dermody

Last updated by author(s): Apr 9, 2023

Reporting Summary

Nature Portfolio wishes to improve the reproducibility of the work that we publish. This form provides structure for consistency and transparency in reporting. For further information on Nature Portfolio policies, see our Editorial Policies and the Editorial Policy Checklist.

Statistics

For all statistical analyses, confirm that the following items are present in the figure legend, table legend, main text, or Methods section.

n/a	Confirmed	
☐	☒	The exact sample size (*n*) for each experimental group/condition, given as a discrete number and unit of measurement
☐	☒	A statement on whether measurements were taken from distinct samples or whether the same sample was measured repeatedly
☐	☒	The statistical test(s) used AND whether they are one- or two-sided<br>*Only common tests should be described solely by name; describe more complex techniques in the Methods section.*
☒	☐	A description of all covariates tested
☒	☐	A description of any assumptions or corrections, such as tests of normality and adjustment for multiple comparisons
☐	☒	A full description of the statistical parameters including central tendency (e.g. means) or other basic estimates (e.g. regression coefficient) AND variation (e.g. standard deviation) or associated estimates of uncertainty (e.g. confidence intervals)
☐	☒	For null hypothesis testing, the test statistic (e.g. *F*, *t*, *r*) with confidence intervals, effect sizes, degrees of freedom and *P* value noted<br>*Give P values as exact values whenever suitable.*
☒	☐	For Bayesian analysis, information on the choice of priors and Markov chain Monte Carlo settings
☒	☐	For hierarchical and complex designs, identification of the appropriate level for tests and full reporting of outcomes
☒	☐	Estimates of effect sizes (e.g. Cohen's *d*, Pearson's *r*), indicating how they were calculated

Our web collection on statistics for biologists contains articles on many of the points above.

Software and code

Policy information about availability of computer code

Data collection

Flow cytometry: FACSDiva™ Software (BD Biosciences, v6.1.3);
Immunoflourescence assay: Gen5 software (BioTek, v3.12);
Atomic force microscopy: Nanoscope analysis software (Bruker, v1.9)
Immunoblotting: Image Studio software (Li-Cor Biosciences, v5.2);
Magnetic resonance imaging: ParaVision (Bruker, v5.1);

Data analysis

Next-generation sequencing: CaRpools (v3.3.2)
Flow cytometry: FlowJo software (v10.8.1);
Immunoflourescence assay: Gen5 software (BioTek, v3.12);
Atomic force microscopy: Origin software (v2022b) and ImageJ (v1.52e);
Immunoblotting: Image Studio Lite software (Li-Cor Biosciences, v5.2);
Magnetic resonance imaging: ITK-SNAP software (v3.8.0);
Data pairwise comparison: Graphpad Prism (v9.5.1).

For manuscripts utilizing custom algorithms or software that are central to the research but not yet described in published literature, software must be made available to editors and reviewers. We strongly encourage code deposition in a community repository (e.g. GitHub). See the Nature Portfolio guidelines for submitting code & software for further information.

Data

Policy information about availability of data

All manuscripts must include a data availability statement. This statement should provide the following information, where applicable:

- Accession codes, unique identifiers, or web links for publicly available datasets
- A description of any restrictions on data availability
- For clinical datasets or third party data, please ensure that the statement adheres to our policy

The data underlying Figs. 1b; 2b-f; 3b-c; 4b-e, h-k; 5; 6; and Supplemental Figs. 3b; 4; 5; 6 are provided as a Source Data file. All other relevant data are available from the corresponding authors on reasonable request.

Human research participants

Policy information about studies involving human research participants and Sex and Gender in Research.

Reporting on sex and gender	N/A
Population characteristics	N/A
Recruitment	N/A
Ethics oversight	N/A

Note that full information on the approval of the study protocol must also be provided in the manuscript.

Field-specific reporting

Please select the one below that is the best fit for your research. If you are not sure, read the appropriate sections before making your selection.

☒ Life sciences ☐ Behavioural & social sciences ☐ Ecological, evolutionary & environmental sciences

For a reference copy of the document with all sections, see nature.com/documents/nr-reporting-summary-flat.pdf

Life sciences study design

All studies must disclose on these points even when the disclosure is negative.

Sample size	We choose the sample size based on data consistency.<br>Typically, for in vitro analyses, 3-4 biological replicates are sufficient to gather consistent results. Two or three independent experiments were repeated to ensure reproducibility.<br>For in vivo mouse experiments, we used at least 6 mice per treatment at every time point. We collected data from 6-17 mice per treatment and time point.
Data exclusions	No data were excluded from the analyses.
Replication	All attempts at replication were successful.
Randomization	Randomization is not applicable to the in vitro experimental procedures.<br>For the in vivo studies, inoculated mice were randomly euthanized for euthanasia and tissue collection at various intervals.
Blinding	(1) For the inoculation of NspPirB-/- mice for virus titer (figure 6c), virulence (figure 6d), and MRI analysis (figure 6e), researchers were blinded to the genotype of the mice during data collection.<br>(2) Blinding is not applicable for other experimental procedures.

Behavioural & social sciences study design

All studies must disclose on these points even when the disclosure is negative.

Study description	N/A
Research sample	N/A

Sampling strategy	N/A
Data collection	N/A
Timing	N/A
Data exclusions	N/A
Non-participation	N/A
Randomization	N/A

Ecological, evolutionary & environmental sciences study design

All studies must disclose on these points even when the disclosure is negative.

Study description	N/A
Research sample	N/A
Sampling strategy	N/A
Data collection	N/A
Timing and spatial scale	N/A
Data exclusions	N/A
Reproducibility	N/A
Randomization	N/A
Blinding	N/A

Did the study involve field work? ☐ Yes ☒ No

Field work, collection and transport

Field conditions	N/A
Location	N/A
Access & import/export	N/A
Disturbance	N/A

Reporting for specific materials, systems and methods

We require information from authors about some types of materials, experimental systems and methods used in many studies. Here, indicate whether each material, system or method listed is relevant to your study. If you are not sure if a list item applies to your research, read the appropriate section before selecting a response.

Materials & experimental systems

n/a	Involved in the study
☐	☒ Antibodies
☐	☒ Eukaryotic cell lines
☒	☐ Palaeontology and archaeology
☐	☒ Animals and other organisms
☒	☐ Clinical data
☒	☐ Dual use research of concern

Methods

n/a	Involved in the study
☒	☐ ChIP-seq
☐	☒ Flow cytometry
☐	☒ MRI-based neuroimaging

Antibodies

Antibodies used

anti-Cas9 mouse mAb 7A9 (Biolegend, #844302);
anti-PirB/A rat mAb 6C1 (Biolegend #144101);
rabbit polyclonal reovirus-specific antiserum (in-house);
anti-myc mouse mAb 9B11 (Cell signaling #2276);
Phospho-Tyrosine (P-Tyr-1000) MultiMab™ Rabbit mAb mix (Cell signaling, #8954S);
anti-GAPDH mouse mAb 71.1 (sigma aldrich #G8795);
Alexa488-conjugated goat rabbit IgG-specific secondary antibody (Invitrogen, #A-11008);
IRDye 680RD goat rabbit IgG-specific IgG (Li-Cor Biosciences, #926-68071);
IRDye 800CW goat mouse IgG-specific IgG (Li-Cor Biosciences, #926-32210).

Validation

The reovirus-specific antiserum was raised in rabbits in a contractual arrangement by our lab and has been used in many of our previous studies, such as DOI: 10.1128/jvi.00055-22.

The other antibodies were purchased commercially and validated at the source. The application of commercial antibodies has been well-validated and cited repeatedly. Relevant validation results and citations can be found in the corresponding commercial web pages listed below.

(1) anti-Cas9 mouse mAb 7A9 (https://www.biolegend.com/en-us/products/purified-anti-crispr-cas9-antibody-11774)
(2) anti-PirB/A rat mAb 6C1 (https://www.biolegend.com/en-us/products/purified-anti-mouse-pir-a-b-antibody-7853)
(3) anti-myc mouse mAb 9B11 (https://www.cellsignal.com/products/primary-antibodies/myc-tag-9b11-mouse-mab/2276)
(4) Phospho-Tyrosine (P-Tyr-1000) MultiMab™ Rabbit mAb mix (https://www.cellsignal.com/products/primary-antibodies/phospho-tyrosine-p-tyr-1000-multimab-rabbit-mab-mix/8954)
(5) anti-GAPDH mouse mAb 71.1 (https://www.sigmaaldrich.com/US/en/product/sigma/g8795)
(6) Alexa488-conjugated goat rabbit IgG-specific secondary antibody (https://www.thermofisher.com/antibody/product/Goat-anti-Rabbit-IgG-H-L-Cross-Adsorbed-Secondary-Antibody-Polyclonal/A-11008)
(7) IRDye 680RD goat rabbit IgG-specific IgG (https://www.licor.com/bio/reagents/irdye-680rd-goat-anti-rabbit-igg-secondary-antibody?utm_source=google&utm_medium=adwords&utm_content=reagent-webpage&utm_campaign=reagents&gclid=Cj0KCQjw_r6hBhDdARIsAMIDhV_PDx6sDfdf9lJSrNogPDYmkAZg6IXwy7f6paqBdrAGEgj-teq8H9EaAnqyEALw_wcB)
(8) IRDye 800CW goat mouse IgG-specific IgG (https://www.licor.com/bio/reagents/irdye-800cw-goat-anti-mouse-igg-secondary-antibody?utm_source=google&utm_medium=adwords&utm_content=reagent-webpage&utm_campaign=reagents&gclid=Cj0KCQjwxMmhBhDJARIsANFGOStJo_cayE1TbdlDwZnC-1ghgeYr6JxF3XfrTevfMb5bfK-UWcZzr88aAsx1EALw_wcB)

Eukaryotic cell lines

Policy information about cell lines and Sex and Gender in Research

Cell line source(s)

Lenti-X 293T cells (Clontech, #632180)
CHO cells (ATCC, CCL-61)
CHO-Lec2 cells (ATCC, CRL-1736)
L929 cells (ATCC, CCL-1)
Primary murine cortex neurons (in house)
Murine embryonic fibroblasts (in house)

Authentication

Use of these cell lines was approved by the Institutional Biosafety Committee of the University of Pittsburgh (Protocol #201600038).
CHO and CHO-Lec2 cells for cDNA transfection and reovirus binding and infection.
L929 cells for reovirus propagation and titer determination.
Primary neurons were isolated from murine embryonic brains and infected with reovirus.
MEFs were isolated from murine embryos and immortalized for CRISPR screening.
Lenti-X 293T cells were used for lentivirus packaging.

Mycoplasma contamination

All cell lines were tested and found to be mycoplasma-negative.

Commonly misidentified lines
(See ICLAC register)

No commonly misidentified cell lines were used in the study

Animals and other research organisms

Policy information about studies involving animals; ARRIVE guidelines recommended for reporting animal research, and Sex and Gender in Research

Laboratory animals	PirB+/+ and PirB-/-: C57BL/6J x 129S4/SvJaeJ (B6 x 129sv) hybrid mice;<br>nestin-cre mice: C57BL/6J mice;<br>PirBfl/fl and neural-specific PirB-/-: C57BL/6J x 129S4/SvJaeJ (B6 x 129sv) hybrid mice.<br><br>Mice over 6 weeks were used for breeding to maintain mouse colonies or prepare neonatal mice for virus inoculation;<br>Neonatal mice (2-3 day) were used for virus inoculation (2-3 day);<br>Primary embryonic fibroblasts or cerebral cortical neurons were isolated from mouse fetuses (E15.5).<br><br>All mice used in this study were maintained in a specific pathogen-free vivarium at the University of Pittsburgh. Mice were inoculated with reovirus in an animal biosafety level 2 (ABSL2) facility. All mice were maintained at a macroenvironmental temperature range of 68 to 76°F (20 to 24.4°C), a relative humidity range of 35% to 55%, and a 12 h/12 h light/dark cycle.
Wild animals	No wild animals were used in the study
Reporting on sex	Information about sex of the mice was collected before euthanasia. Mice of both sexes in equal proportion were used in these experiments. Since there is no evidence that sex of the mice influences experimental outcomes, we conducted all of the analyses in this study without consideration of mouse sex.
Field-collected samples	No field-collected samples were used in the study
Ethics oversight	All animal husbandry and experimental procedures were conducted in accordance with U.S. Public Health Service policy and approved by the Institutional Animal Care and Use Committee at the University of Pittsburgh.

Note that full information on the approval of the study protocol must also be provided in the manuscript.

Flow Cytometry

Plots

Confirm that:

☒ The axis labels state the marker and fluorochrome used (e.g. CD4-FITC).

☒ The axis scales are clearly visible. Include numbers along axes only for bottom left plot of group (a 'group' is an analysis of identical markers).

☒ All plots are contour plots with outliers or pseudocolor plots.

☒ A numerical value for number of cells or percentage (with statistics) is provided.

Methodology

Sample preparation	MEFs were tranduced with CRISPR library-encoding lentiviruses and selected for antibiotic-resistance cells.<br>CHO cells were transfected with cDNA. MEFs and CHO cells were disassociated with Cellstripper and bound with fluoresence-labelled reovirus and mAb. CHO cells were fixed with paraformaldehyde before flow cytometry analysis by LSRII. Live MEFs were sorted with FACSAria IIu.
Instrument	BD LSRII and BD FACSAria IIu
Software	BD FACSDiva; FlowJo
Cell population abundance	Reovirus binding assay/receptor expression: 50,000 events per sample.<br>Library-transduced MEFs: 5-10 million events per sample.
Gating strategy	SSC/FFC gating is determined empirically; fluorescence gating is based on positive and negative controls.

☒ Tick this box to confirm that a figure exemplifying the gating strategy is provided in the Supplementary Information.

Magnetic resonance imaging

Experimental design

Design type	In vivo multi-planar T2-weighted anatomical MRI covering the entire brain
Design specifications	T2-weighted MRI covering the entire mouse brain was used to visualize foci with edema resulting from inflammation. Edema with longer T2 appears as hyperintenity (brightness) on the T2-weighted images. In contrast, hemorrhage due to injection appears as hypointensity (darkness).

Behavioral performance measures

a. MRI acquisition: An MRI operator blinded to the mouse genotype and infection status conducted the MRI acquisition using an identical protocol for all mice. The mice from the knockout test group, the wild-type control group, and the sham controls were numbered in a way that their groups were not identifiable by the MRI operator. The order of MRI scanning was randomized so that the mouse identity was not identifiable by the MRI operator.

b. Animal procedure for MRI acquisition: All mice were subjected to an identical anesthesia and MRI protocol. Respiration was monitored using a pneumatic sensor placed between the animal bed and the mouse's diaphragm while body temperature was measured using a fiber optic sensor and maintained with feedback-controlled warm air source. The overall scan time for each animal was comparable.

c. MRI analysis: The volumes of hyperintensity (reflecting edema resulting from inflammation) and hypointensity (reflecting hemorrhage due to injection) were manually segmented by an analyzer blinded to the identity of the animals. The order of analysis was randomized.

Acquisition

Imaging type(s)

In vivo multi-planar T2-weighted anatomical MRI

Field strength

7-Tesla field strength

Sequence & imaging parameters

slice number = 15, field of view (FOV) = 1.8 cm, matrix = 256 X 256, slice thickness = 0.65 mm, in-plane resolution = 70 µm, echo time (TE) = 12 msec, RARE factor = 8, effective echo time (TE) = 48 msec, repetition time (TR) = 1551.2 msec, and flip angle (FA) = 180 degree.

Area of acquisition

Entire brain with both cerebral hemispheres. Multi-planar consecutive axial slices without gaps covering from the posterior end of the olfactory bulb to the cerebellum.

Diffusion MRI ☐ Used ☒ Not used

Preprocessing

Preprocessing software

a. MRI signals were Fourier-transformed, reconstructed, and exported to the DICOME format using Bruker ParaVision 5.1 software.
b. The DICOM data were analyzed by an analyzer blinded to the study using the open source ITK-SNAP brain segmentation software.

Normalization

To account for potential differences in brain sizes between infected animals and sham controls, the volumes of hyperintensity or hypointensity were normalized to the entire brain volume of each animal.

Normalization template

NA

Noise and artifact removal

No noise nor artifact removal was performed. All analyses were conducted using the original raw data.

Volume censoring

No volume censoring was performed. All the volumetric data reported are original raw measurements. During MRI acquisition, all mouse heads were secured inside the coil to eliminate motion artifact during acquisition, thus there is no significant motion artifact present in our raw data.

Statistical modeling & inference

Model type and settings

No fMRI nor modeling performed.

Effect(s) tested

NA

Specify type of analysis: ☒ Whole brain ☐ ROI-based ☐ Both

Statistic type for inference (See Eklund et al. 2016)

NA

Correction

NA

Models & analysis

n/a	Involved in the study
☒	☐ Functional and/or effective connectivity
☒	☐ Graph analysis
☒	☐ Multivariate modeling or predictive analysis
